# Repeatability Using Automatic Tracing with Canon OCT- HS100 and Zeiss Cirrus HD-OCT 5000

**DOI:** 10.1371/journal.pone.0149138

**Published:** 2016-02-11

**Authors:** Rune Brautaset, Ulrika Birkeldh, Petra Frehr Alstig, Petra Wikén, Maria Nilsson

**Affiliations:** Unit of Optometry, Department of Clinical Neuroscience, St. Erik Eye Hospital, Karolinska Institutet, Stockholm, Sweden; Stanford University Medical Center, UNITED STATES

## Abstract

**Background:**

Optical coherence tomography (OCT), can be used in clinical practice to provide high resolution cross-sectional images of the retina, optic disc and macula structure. These measurements can be useful for early detection, diagnosis, monitoring and treatment guidance for retinal diseases. Therefore, repeatability of measurements in OCT is of great importance.

**Methods:**

Macula and optic disc parameters from the right eye of 30 healthy subjects were obtained twice with the Canon OCT-HS100 and Zeiss Cirrus HD-OCT 5000. Repeatability was evaluated by use of the coefficient of repeatability (CR) and the coefficient of repeatability as a percentage of the mean (CR%), and the obtained measurements were compared between the instruments.

**Results:**

CR% of optic disc parameters ranged between 0.90 and 22.22% and 0.00 and 16.00% with the Canon and Zeiss OCT respectively. For macular parameters CR% ranged between 0.62 and 2.81% and 0.99 and 1.81% with the Canon and Zeiss OCT respectively. No statistical difference could be found when comparing the CR of all macular and disc measurements between the instruments. Compared to our previously published data repeatability has significantly improved with the inclusion of automatic tracking systems with both the Canon and Zeiss OCT.

**Conclusion:**

Automatic tracking function improves repeatability in both Canon OCT-HS100 and Zeiss Cirrus HD-OCT 5000. However, measurements generated by the two instruments are still not interchangeable.

## Introduction

Optical coherence tomography (OCT), is used in clinical practice to provide high resolution cross-sectional images of the retina, optic disc and macula. Each of the retinas’ distinctive layers can be seen, allowing the clinician to map and measure their thickness. These measurements can be useful for early detection, diagnosis, monitoring and treatment guidance for retinal diseases, such as age related macular degeneration [1,2], glaucoma [3–5] and multiple sclerosis [6–8].

OCT measurement is a non-invasive, non-contact imaging system and provides instant imaging. In comparison to, e.g., ultrasound it gives higher image resolution [9]. The measurements do not require pupil dilation in order to produce high image quality [10–12].

The test time is rather short, but may require the patient's cooperation in terms of not blinking and maintaining a steady fixation. To improve measurements and simplify the procedure for both patients and operators, some suppliers have incorporated automatic retinal tracking system.

Langnegger et al. [13] (using the Heidelberg Spectralis SD-OCT) showed that the repeatability was good in both normal and glaucomatous eyes and could be significantly improved by using an automatic tracing system. These findings suggested that automatic tracking was capable of significantly improving the repeatability of retinal nerve fiber (RNFL) thickness measurements due to automatic re-scanning when fixation errors and blinks occurred [13]. In general the repeatability of SD-OCT measurements have been found to be high [14], however, differences between instruments can be caused by differences in the algorithms used to detect retinal structural layers [15,16]. Differences between instruments are therefore likely to occur when comparing segmented images. Retinal diseases, such as epiretinal membranes, will also affect the ability to detect structures and consequently also repeatability [17,18].

In a previously published paper we [19] evaluated and compared the repeatability of disc and macula OCT measurements without the use of automatic eye tracking with the Canon OCT-HS100 and Zeiss Cirrus-OCT 4000. In newer models of both instruments (software upgrade in the Canon and version 5000 of the Zeiss OCT) automatic tracing systems are available. During scanning, both OCTs automatically track the retina which makes the scanning procedure immediately recover in case of eye movements or blinking, i.e., an additional scan is automatically performed to correct the error. Since this function prevents re-taking scans due to eye movement it saves time in a clinical setting. With this feature, OCT measurements can be less operator dependent. It also makes it easier to obtain scans from patients who have difficulties maintaining a steady fixation (e.g., children or patients with impaired fixation ability due to maculopathies) [20]. Due to the ability to recollect the exact same location in the retinal tissue as in previous examination, it enhances comparison and monitoring progression of pathologies in the retina.

The purpose of the study was to evaluate the repeatability of OCT measurements in healthy eyes obtained with the Canon OCT-HS100 (version 4.00) and the Zeiss Cirrus HD-OCT 5000 (version 8.0) with the use of their incorporated automatic tracking systems.

## Methods

Thirty consecutive subjects, without ocular pathologies, in whom well-segmented OCT images could be obtained, were included in the study. The mean age was 35.2 years (range 22–66) and the female/male ratio was 24/6. Scans with the Cirrus HD-OCT 5000 (version 8.0) were obtained in the Open Clinic at the Unit of Optometry, St. Erik Eye Hospital, Stockholm, Sweden and with the Canon OCT–HS100 (version 4.00) at the nearby Neurology department at the Karolinska Hospital. Only the right eye of each subject was included. The measurements were performed without the use of mydriatic drops [10–12]. The study was approved by the regional ethics committee in Stockholm (Regionala Etikprovningsnämnden i Stockholm) and performed in accordance with the ethical standards stated in the Declaration of Helsinki. Each subject signed an informed consent before the enrolment in the study.

Exclusion criteria were poor OCT image quality (signal strength ≤7), presence of media opacities, epiretinal membrane or vitreomacular traction, and a history of ocular trauma.

The Canon OCT-HS100 has an A-scan velocity of 70,000 scans/sec with a claimed axial resolution of 3 μm and a scanning depth of 2 mm. The scanning width is between 2 and 10 mm and the wavelength used is 855 nm. With the Canon OCT-HS100 a Disc 3D and a Macula 3D scans were obtained. The customized 3D scan for evaluation of the optic nerve head (Disc 3D) is acquired over a 6X6 mm area by 256 B-scans each composed of 512 A-scans. The macular cube measurement (Macula 3D) is built up from 128 B-scans each composed of 1024 A-scans within a 10X10 mm area.

The Cirrus HD-OCT 5000 has an A-scan velocity of 27,000 scans/sec with a 5 μm axial resolution and a scanning depth of 2 mm. The instrument uses light of 840nm wavelength and scans an area of 6X6 mm for both macula and disc scans. With the Cirrus HD-OCT the Optic Disc and Macular Cubes were obtained. The Optic Disc Cube acquires 200 B-scans, each composed of 200 A-scans. The Macular Cube acquires 128 B-scans each composed of 512 A-scans. With both instruments all scans obtained were reviewed to assert that segmentation was correct before analysis was done. All scans were obtained twice with the subjects being re-seated in front of the instrument before the second scan.

Only equivalent parameters obtained by both instruments were used for analysis. For optic disc measurements disc area, rim area, cup volume, vertical cup/disc (C/D) ratio, retinal nerve fibre layer (RNFL) average thickness and RNFL thickness in the four quadrants of the optic nerve head (i.e., according to ISNT–inferior, superior, nasal and temporal) were used. For macular measurements the nine subfields corresponding to the Early Treatment of Diabetic Retinopathy Study (EDTRS) [21] areas were used. ETDRS areas are defined by three concentric rings centred into the fovea with diameters of 1, 3 and 6 mm lines.

The main objective was to evaluate macula and optic disc measurement repeatability using the automatic tracing function in the Canon OCT-HS100 and the Zeiss Cirrus HD-OCT 5000 and to evaluate whether the function improved the repeatability in comparison to the study of Brautaset et al. [19].

## Results

The coefficient of repeatability (CR) was calculated according to the methods outlined by Bland and Altman [22] for each of the optic disc and macular thickness parameters (1.96 x square root of the within-subject variance from repeated measurements). Also the coefficient of repeatability as a percentage of the mean (CR%) was calculated (CR% = (CR/mean)*100).

The squared Pearson correlation coefficient (R^2^) was also calculated for each of the measurements and the difference between the first and the second measurement statistically evaluated to see if it differed from zero. For comparison between the two instruments statistical comparison of the CR values were done, alongside squared Pearson correlation (R^2^). In all thirty subjects scans with signal strength ≥7 were obtained. For analysis of vertical C/D ratio one subject was excluded since one instrument estimated the C/D ratio to be zero which is physiologically impossible.

Average results for all the optic disc and macular thickness parameters can be seen in Tables 1 and 2 respectively together with statistical analysis of the difference between the first and second measurement with each instrument (one-sample t test or Wilcoxon rank-sum test dependent on distribution). In Table 3 the mean difference (Canon-Zeiss) for each disc and macular parameter can be seen together with statistical evaluation of between instrument comparisons (One-way analysis of variance with post-hoc test). For all statistical analysis statistical significance threshold was set to P < 0.05.

**Table 1:** Repeatability of optic disc parameters.

Optic disc parameters (within instruments)										
	Canon OCT-HS100					Zeiss Cirrus HD-OCT				
	Mean	CR	CR%	R^2^	p	Mean	CR	CR%	R^2^	p
Disc area (mm^2^)	2.08 ±0.41	0.21	10.10	0.92	0.61^t^	1.87 ±0.35	0.10	5.34	0.96	0.71^t^
Rim area (mm^2^)	1.58 ±0.37	0.20	12.66	0.97	0.55^t^	1.41 ±0.24	0.08	5.67	0.97	0.97^t^
Cup volume (mm^3^)	0.09 ±0.12	0.02	22.22	0.98	0.63^w^	0.13 ±0.12	0.02	16.00	0.99	0.71^t^
C/D vertical ratio	0.48 ±0.14	0.05	10.41	0.97	0.99^w^	0.47 ±0.12	0.04	8.51	0.96	0.45^t^
RNFL average (μm)	99.16 ±8.57	5.46	5.51	0.93	0.15^w^	93.96 ±8.95	3.39	3.61	0.96	0.21^t^
RNFL Inferior (μm)	127.80 ±36.47	1.15	0.90	0.92	0.71^t^	122.86 ±34.41	1.09	0.89	0.96	0.61^t^
RNFL Superior (μm)	118.36 ±31.43	2.27	1.92	0.76	0.16^t^	112.57 ±30.29	0.89	0.79	0.94	0.68^t^
RNFL Nasal (μm)	80.52 ±18.94	2.09	2.60	0.89	0.15^t^	72.23 ±17.33	0.00	0	0.98	0.99^t^
RFNL Temporal (μm)	69.60 ±16.03	1.50	2.16	0.91	0.35^t^	65.11 ±16.24	0.89	1.37	0.97	0.53^t^

(C/D = cup/disc; CR = coefficient of repeatability; CR% = coefficient of repeatability as a percentage of mean; RNFL = Retinal nerve fiber layer.

t = one- sample t test / w = Wilcoxon rank-sum test)

**Table 2:** Repeatability of macular parameters.

Macular parameters (within instruments)										
	Canon OCT-HS100					Zeiss Cirrus HD-OCT				
Thickness (μm)	Mean	CR	CR%	R^2^	p	Mean	CR	CR%	R^2^	p
Centre	276.22 ±15.69	5.04	1.82	0.97	0.95^t^	259.34 ±15.97	2.57	0.99	0.99	0.55^w^
Inner superior	351.08 ±12.07	2.82	0.80	0.98	0.99^t^	324.82 ±11.48	5.25	1.62	0.95	0.36^t^
Inner nasal	352.02 ±14.13	3.11	0.88	0.99	0.07^w^	327.27 ±13.72	4.28	1.31	0.97	0.36^t^
Inner inferior	347.33 ±10.99	4.46	1.28	0.95	0.71^t^	322.38 ±11.99	4.05	1.26	0.97	0.58^t^
Inner temporal	335.34 ±10.82	2.09	0.62	0.99	0.90^w^	311.41 ±10.57	4.20	1.35	0.96	0.15^t^
Outer superior	305.21 ±12.17	8.58	2.81	0.89	0.16^t^	277.65 ±12.42	6.65	1.67	0.96	0.38^t^
Outer nasal	317.66 ±14.57	2.98	0.94	0.99	0.29^t^	295.48 ±14.24	3.57	1.21	0.98	0.44^t^
Outer inferior	290.74 ±12.22	4.80	1.65	0.97	0.35^t^	267.27 ±14.21	3.73	1.40	0.98	0.26^t^
Outer temporal	287.54 ±10.17	4.00	1.39	0.96	0.69^t^	258.96 ±9.55	4.70	1.81	0.93	0.50^t^

Repeatability of macular parameters obtained with the Canon OCT-HS100 and Zeiss Cirrus HD-OCT 5000. (C/D = cup/disc; CR = coefficient of repeatability; CR% = coefficient of repeatability as a percentage of mean. t = one- sample t test. w = Wilcoxon rank-sum test)

**Table 3:** Between instrument comparison.

Between instrument comparison		
*Optic disc*	Mean difference (p-value)	R^2^ (p-value)
Disc area (mm^2^)	0.22 (p>0.05)	0.80 (p<0.05)
Rim area (mm^2^)	0.17(p>0.05)	0.73 (p<0.05)
Cup volume (mm^3^)	-0.03 (p>0.05)	0.97 (p<0.05)
C/D vertical ratio	-0.03 (p>0.05)	0.85 (p<0.05)
RNFL average (μm)	5.20 (p>0.05)	0.87 (p<0.05)
RNFL Inferior (μm)	5.36 (p>0.05)	0.82 (p<0.05)
RNFL Superior (μm)	5.43 (p>0.05)	0.67 (p<0.05)
RNFL Nasal (μm)	8.02(p>0.05)	0.69 (p<0.05)
RFNL Temporal (μm)	4.00 (p>0.05)	0.66 (p<0.05)
***Macula***		
Centre (μm)	16.87 (p<0.05)	0.96 (p<0.05)
Inner superior (μm)	26.25 (p<0.05)	0.43 (p<0.05)
Inner nasal (μm)	24.74 (p<0.05)	0.62 (p<0.05)
Inner inferior (μm)	24.95 (p<0.05)	0.53 (p<0.05)
Inner temporal (μm)	23.93 (p<0.05)	0.67 (p<0.05)
Outer superior (μm)	27.55 (p<0.05)	0.59 (p<0.05)
Outer nasal (μm)	22.18 (p<0.05)	0.60 (p<0.05)
Outer inferior (μm)	23.46 (p<0.05)	0.62 (p<0.05)
Outer temporal (μm)	28.58 (p<0.05)	0.64 (p<0.05)

Between instrument comparison of the Canon OCT-HS100 and Zeiss Cirrus HD-OCT 5000, mean difference (Canon-Zeiss) and correlation coefficient (R^2^). (C/D = cup/disc; RNFL = Retinal nerve fibre layer. Positive values denote greater/thicker measurement obtained with the Canon OCT-HS100, and negative values lower/thinner values.)

For comparison of the two instruments, paired t-test was used to evaluate differences in CR values; no statistical difference could be found (t = 0.49; p = 0.63).

## Discussion

This study provides data of the test-retest repeatability using automatic tracking with the Canon OCT-HS100 and Zeiss Cirrus HD-OCT 5000. In general, the measured optic disc and macular values were found to be repeatable with CR% values in optic disc parameters ranging between 0.90 and 22.22%, and between 0.00 and 16.00% with the Canon and Zeiss respectively. Slight differences in repeatability between the instruments can be due to the difference in resolution between the instruments. Lower resolution, as with the Zeiss, may make it easier to replicate the segmentation. However, differences between instruments can also be caused by differences in the algorithms used to define retinal structural layers independent of resolution [15,16]. When excluding cup volume from the analysis, CR% values of the optic disc, range from 0.90 to 12.66% and 0.00–8.51% with the Canon and Zeiss respectively, which is in line with prior studies [19,23–27]. Measurements in the macular region provided good repeatability, better than in the optic disc. This result is probably due to anatomical features, for example the absence of large blood vessels in the macula [14]. The CR% for macular parameters ranged between 0.62 and 2.81%, and 0.99 and 1.81% with the Canon and Zeiss respectively.

The least repeatable measurements were obtained for the cup volume in both instruments. Measuring the cup volume and C/D-ratio has shown to be difficult. This is in agreement with other studies [19,22,26]. In comparison to the study of Brautaset et al. [19] the CR% in macula has increased with the automatic tracking.

In general, all measurements obtained with the Canon OCT are greater/thicker in both the optic disc and macular region with exception of C/D-vertical ratio and cup-volume (Table 3). This can most likely be explained by different scanning circle diameter, where the Canon device uses a slightly smaller scanning circle [19]. In spite of this, the correlation between the two devices is good, but because of the difference in thickness estimation, they are not interchangeable.

Repeatability is of great importance in a clinical setting when monitoring the progression of for example glaucoma. Viewing this result, it seems as if the automatic tracking function makes obtaining measurements less operator dependent.

On the other hand, in the study of Langenegger et al. [13] using Spectralis SD-OCT with a similar eye tracking system the repeatability on glaucoma eyes was significantly higher. This indicates that eye tracking improves repeatability in pathological eyes. If this is the case using Cirrus and Canon needs to be evaluated.

The aim of this study was also to evaluate whether automatic tracking function in Canon and Zeiss OCT has improved the repeatability. In order to analyse this, the CR values from this study were compared with the CR values of the study performed by Brautaset et al. [19]. Statistical analysis found the CR values to significantly lower with the automatic tracing for both the Canon (t = 3.348; p = 0.0038) and Zeiss (t = 4.80; p = 0.0008) OCT.

In clinical practice, a reduction of 4.3 μm of RNFL thickness is considered a clinically significant glaucomatous structural change [28]. The variation of CR in the present study ranges with the Canon from 0.90 to 2.60 μm regarding ISNT-measurements. In Zeiss, the CR ranged between 0.00 and 1.37 μm. This implicates that the instrument variability within ISNT are accurate enough regarding clinical use.

In conclusion the automatic tracking function improves repeatability in both Canon OCT-HS100 and Zeiss Cirrus HD-OCT 5000. However, due to the difference in thickness estimation the measurements made by the two instruments are still not interchangeable.

## Supporting Information

S1 File Raw data first measurement Canon and Zeiss OCT. (PDF)

S2 File Raw data second measurement Canon and Zeiss OCT. (PDF)
