# Supplementary material for: Repeatability Using Automatic Tracing with Canon OCT- HS100 and Zeiss Cirrus HD-OCT 5000
Source: PLoS One. 2016 Feb 11;11(2):e0149138. doi: 10.1371/journal.pone.0149138 (PMC4750906; doi:10.1371/journal.pone.0149138)
Supplement: S1 File — (PDF) [file pone.0149138.s001.pdf]

S1. Raw data first measurment Canon and Zeiss OCT

	Canon															
	ONH Measurements				RFNL Measurement	Macula										
Subject	C Disc area	C Rim Area	C Cup volume	C C/D Verti	C TSNIT average	C Center	C 12--1	C 3--1	C 6--1	C 9--1	C12--2	C 3--2	C 6--2	C 9--2	C Min fovea RPE	
1	2,1	1,5	0,23	0,53	92	286	346	350	348	336	284	290	278	275	226	
2	2,21	1,43	0,12	0,63	88	280	349	353	340	336	313	308	283	292	232	
3	2,1	1,44	0,1	0,55	102	270	337	338	340	321	298	311	283	279	229	
4	1,9	1,68	0,03	0,32	113	288	357	354	353	342	317	331	305	304	243	
5	1,88	1,41	0,09	0,48	97	271	348	343	340	329	303	307	282	286	226	
6	2,02	1,96	0	0,15	97	272	348	344	345	334	302	306	286	277	226	
7	1,52	1,29	0,07	0,35	109	268	344	350	346	329	311	322	299	288	212	
8	2,32	1,59	0,26	0,52	102	268	347	349	352	334	304	328	304	287	219	
9	1,78	1,61	0,02	0,28	88	289	352	358	353	343	287	315	300	282	231	
10	2,13	1,86	0,01	0,33	87	323	338	347	337	340	277	295	269	278	284	
11	1,68	1,36	0,03	0,43	93	284	368	368	357	344	309	327	299	290	222	
12	1,47	1,39	0	0,22	100	255	328	327	328	305	289	296	281	278	232	
13	1,47	0,98	0,11	0,63	86	286	364	364	356	348	307	327	285	293	231	
14	2,57	1,78	0,18	0,58	101	286	350	358	356	334	311	323	300	291	229	
15	2,18	1,33	0,2	0,66	97	281	350	360	343	334	299	314	280	271	212	
16	2,09	1,93	0,01	0,3	101	277	351	354	346	339	315	311	272	276	229	
17	1,67	1,13	0,15	0,52	94	249	333	326	331	320	287	297	275	269	213	
18	2,41	2,13	0,03	0,29	109	244	351	348	342	333	318	326	304	300	197	
19	2,25	1,54	0,12	0,57	100	286	371	377	361	354	320	339	295	302	222	
20	2,63	2,31	0,04	0,42	96	272	349	352	347	339	301	311	288	286	216	
21	2,03	1,39	0,27	0,47	90	258	352	355	349	332	301	316	294	295	211	
22	1,69	1,24	0,11	0,47	98	292	334	341	330	330	290	303	275	279	245	
23	1,79	1,19	0,14	0,64	91	271	341	341	334	323	291	301	274	275	214	
24	2,9	2,54	0,04	0,33	110	271	362	361	352	343	313	332	302	294	216	
25	1,85	1,39	0,1	0,52	96	278	348	347	346	336	301	305	272	288	221	
26	3,18	1,88	0,36	0,65	125	263	369	366	366	349	329	351	321	303	213	
27	1,71	1,14	0,09	0,48	90	300	352	356	351	336	298	323	292	284	253	
28	1,76	1,28	0,08	0,63	99	261	325	318	320	321	282	294	274	274	211	
29	2,12	1,07	0,38	0,68	102	264	341	337	335	325	313	320	294	292	226	
30	1,76	1,28	0,08	0,63	99	261	325	318	320	321	282	294	274	274	211	

Zeiss																	
Disc					Fovea										Age	Borne	Sex
Z RNFL average	Z Rim area	Z Disc area	Z C/D Verti	Z Cup volume	Z Center	Z 12--1	Z 3--1	Z 6--1	Z 9--1	Z 12--2	Z 3--2	Z 6--2	Z 9--2				
88	1,43	1,92	0,51	0,238	268	316	325	322	310	259	270	255	249		22	1992	0
82	1,33	2,09	0,59	0,169	265	321	330	323	314	288	291	268	264		38	1976	1
93	1,34	1,88	0,52	0,127	253	319	317	316	294	275	296	265	249		44	1970	1
105	1,46	1,72	0,32	0,041	270	329	327	327	318	290	310	289	275		47	1967	1
90	1,22	1,58	0,42	0,104	251	321	318	313	303	280	286	257	256		41	1973	1
93	1,7	1,89	0,24	0,01	253	324	323	322	309	275	284	267	249		33	1981	1
102	1,26	1,44	0,33	0,088	248	322	325	323	307	283	301	277	258		26	1988	1
97	1,26	1,91	0,56	0,263	255	332	335	336	321	285	315	291	265		30	1984	1
85	1,53	1,64	0,3	0,02	269	322	325	318	309	255	287	269	249		23	1991	0
82	1,46	1,79	0,44	0,036	307	313	323	314	317	254	273	246	254		66	1948	0
89	1,34	1,58	0,41	0,049	274	343	346	330	321	280	303	269	260		24	1990	1
94	1,48	1,53	0,2	0,008	238	302	300	304	287	259	277	255	246		33	1981	1
83	1,05	1,41	0,5	0,113	264	335	337	334	320	280	302	264	264		25	1989	0
96	1,46	2,19	0,56	0,21	273	326	336	333	312	281	303	272	261		26	1988	1
88	1,15	1,78	0,6	0,223	268	329	337	318	311	275	291	256	246		62	1952	1
98	1,73	1,84	0,27	0,014	264	332	336	324	320	272	294	253	252		66	1948	1
89	1,17	1,61	0,49	0,197	235	311	304	307	297	267	279	253	251		24	1990	1
99	1,92	2,25	0,35	0,051	232	331	333	327	315	291	314	291	272		22	1992	1
94	1,3	1,98	0,58	0,149	268	341	349	332	326	289	310	262	268		51	1963	1
86	1,49	1,78	0,45	0,042	257	328	335	330	314	278	298	273	254		26	1988	1
89	1,21	1,8	0,52	0,296	238	329	333	327	313	283	302	263	262		26	1988	1
92	1,15	1,5	0,42	0,13	269	307	318	308	306	271	286	253	251		44	1970	0
87	1,18	1,64	0,58	0,158	257	315	318	312	302	260	279	256	250		45	1969	1
110	1,95	2,44	0,46	0,036	252	329	328	319	312	284	299	274	264		24	1990	1
89	1,28	1,87	0,51	0,155	256	318	320	322	307	271	285	254	260		24	1990	1
123	1,95	3,16	0,58	0,397	251	347	351	356	333	305	335	309	284		26	1988	1
86	1,19	1,73	0,51	0,145	281	328	334	326	311	273	301	271	260		44	1970	0
90	1,16	1,64	0,53	0,117	243	300	297	298	297	263	280	252	246		35	1979	1
102	1,18	2,12	0,65	0,442	243	311	304	302	294	285	299	267	263		23	1991	1
90	1,16	1,64	0,53	0,117	243	300	297	298	297	263	280	252	246		35	1979	1
