# Supplementary material for: Repeatability Using Automatic Tracing with Canon OCT- HS100 and Zeiss Cirrus HD-OCT 5000
Source: PLoS One. 2016 Feb 11;11(2):e0149138. doi: 10.1371/journal.pone.0149138 (PMC4750906; doi:10.1371/journal.pone.0149138)
Supplement: S2 File — (PDF) [file pone.0149138.s002.pdf]

S2. Raw data second measurment Canon and Zeiss OCT

	Canon															
	ONH Measurements				RFNL Measurement	Macula										
Subject	C Disc area	C Rim Area	C Cup volume	C C/D Verti	C TSNIT average	C Center	C 12--1	C 3--1	C 6--1	C 9--1	C12--2	C 3--2	C 6--2	C 9--2	C Min fovea RPE	
1	2,08	1,48	0,22	0,52	92	284	344	349	346	336	285	289	276	277	226	
2	2,2	1,45	0,12	0,62	91	280	348	352	341	336	311	308	284	291	239	
3	2,16	1,51	0,1	0,54	99	269	337	335	339	320	296	314	282	273	229	
4	1,97	1,73	0,03	0,35	106	285	356	353	351	342	316	331	306	305	240	
5	1,85	1,4	0,09	0,47	99	272	346	343	339	329	305	306	279	286	226	
6	2	1,93	0	0,14	97	274	349	344	344	334	303	307	285	276	226	
7	1,91	1,68	0,07	0,28	107	268	343	351	347	329	310	323	300	288	214	
8	2,08	1,38	0,23	0,53	101	267	346	348	351	334	302	327	304	287	216	
9	1,83	1,68	0,02	0,28	88	289	354	358	355	343	288	316	298	281	233	
10	2,14	1,85	0,01	0,34	85	323	339	348	336	338	277	297	267	275	281	
11	1,69	1,37	0,03	0,45	92	282	366	365	354	344	310	328	296	288	219	
12	1,54	1,46	0	0,23	100	253	326	324	327	306	288	298	279	275	229	
13	1,49	0,98	0,11	0,61	84	285	364	363	356	348	311	326	283	292	227	
14	2,43	1,69	0,17	0,57	102	289	350	360	358	333	313	322	298	294	229	
15	2,08	1,28	0,2	0,66	98	281	350	360	344	334	299	313	282	273	213	
16	2,07	1,92	0,01	0,26	108	278	350	352	343	339	293	312	268	272	227	
17	1,81	1,28	0,15	0,49	93	250	334	328	332	322	284	298	273	270	215	
18	2,5	2,2	0,04	0,29	106	248	353	349	341	332	320	325	308	303	201	
19	2,17	1,48	0,12	0,58	97	291	373	379	366	355	322	340	298	304	223	
20	2,47	2,12	0,04	0,43	95	272	349	350	346	338	301	311	287	285	216	
21	2,02	1,36	0,27	0,49	89	250	349	353	352	334	303	317	287	293	208	
22	1,68	1,27	0,1	0,43	99	290	337	344	338	333	291	308	279	279	245	
23	1,82	1,24	0,13	0,6	91	271	340	340	333	323	288	300	275	276	214	
24	2,91	2,56	0,03	0,33	110	271	362	360	351	343	313	330	302	295	218	
25	1,89	1,35	0,12	0,56	97	273	347	346	346	335	299	306	275	288	219	
26	3,34	1,95	0,4	0,67	123	266	369	367	368	351	330	351	320	303	214	
27	1,75	1,19	0,09	0,46	89	299	352	357	351	336	298	325	291	282	254	
28	1,76	1,3	0,08	0,58	98	259	324	318	320	320	281	294	274	274	212	
29	2,08	1,02	0,37	0,71	102	264	343	336	334	325	314	319	293	291	223	
30	1,89	1,35	0,12	0,56	97	273	347	346	346	335	299	306	275	288	219	

Zeiss														
Disc					Fovea									
Z RNFL average	Z Rim area	Z Disc area	Z C/D Verti	Z Cup volume	Z Center	Z 12--1	Z 3--1	Z 6--1	Z 9--1	Z 12--2	Z 3--2	Z 6--2	Z 9--2	
88	1,41	1,92	0,55	0,25	270	317	326	323	311	260	270	256	249	
84	1,34	2,1	0,56	0,167	268	330	336	326	319	294	293	269	271	
91	1,3	1,82	0,52	0,122	254	318	319	319	295	274	297	268	251	
105	1,44	1,68	0,33	0,04	270	330	328	326	317	292	310	284	276	
87	1,26	1,62	0,4	0,105	253	324	322	316	305	282	288	258	256	
93	1,68	1,89	0,24	0,011	254	328	324	326	312	280	287	270	255	
101	1,23	1,43	0,35	0,096	249	322	326	323	307	283	300	277	258	
99	1,33	2,01	0,53	0,268	253	329	332	333	316	284	312	289	261	
85	1,55	1,67	0,31	0,019	270	325	325	317	309	256	287	269	250	
81	1,45	1,77	0,44	0,035	308	314	323	314	319	254	275	246	254	
91	1,35	1,6	0,42	0,052	275	345	347	330	322	283	303	270	262	
94	1,5	1,55	0,18	0,007	237	303	301	304	287	259	277	256	246	
82	1,08	1,45	0,51	0,114	265	336	337	331	320	281	302	262	263	
97	1,37	2,08	0,56	0,201	273	328	338	335	315	284	307	274	261	
91	1,12	1,73	0,59	0,214	267	329	336	319	312	277	291	257	246	
95	1,72	1,84	0,3	0,015	264	332	336	323	319	274	294	250	253	
89	1,25	1,67	0,46	0,168	234	308	304	306	296	265	280	254	246	
101	1,88	2,23	0,37	0,053	231	328	328	323	311	289	310	288	270	
96	1,3	1,96	0,57	0,152	269	344	351	334	329	290	311	265	268	
87	1,43	1,67	0,44	0,036	256	324	334	330	314	275	297	274	256	
89	1,23	1,8	0,47	0,274	235	326	329	324	311	279	297	263	263	
97	1,19	1,52	0,42	0,117	270	308	319	309	308	272	286	253	252	
86	1,21	1,7	0,58	0,164	258	317	319	314	304	263	280	256	252	
110	1,96	2,44	0,46	0,037	251	329	328	318	312	281	300	274	261	
89	1,18	1,73	0,54	0,147	256	320	319	319	307	273	284	251	259	
123	1,94	3,15	0,6	0,393	253	348	353	358	336	308	335	307	286	
87	1,24	1,75	0,46	0,13	280	326	331	324	309	272	300	270	258	
91	1,15	1,63	0,54	0,116	243	302	297	297	297	265	280	252	245	
104	1,25	2,19	0,67	0,442	243	309	305	304	296	286	300	268	263	
89	1,18	1,73	0,54	0,147	256	320	319	319	307	273	284	251	259	
